# CPUY201112, a novel synthetic small-molecule compound and inhibitor of heat shock protein Hsp90, induces p53-mediated apoptosis in MCF-7 cells

**DOI:** 10.1038/srep19004

**Published:** 2016-01-08

**Authors:** Xiao-Li Xu, Qi-chao Bao, Jian-Min Jia, Fang Liu, Xiao-Ke Guo, Ming-ye Zhang, Jin-lian Wei, Meng-chen Lu, Li-li Xu, Xiao-Jin Zhang, Qi-Dong You, Hao-Peng Sun

**Affiliations:** 1 Jiangsu Key Laboratory of Drug Design and Optimization, China Pharmaceutical University, Nanjing, 210009, China; 2 Department of Medicinal Chemistry, School of Pharmacy, China Pharmaceutical University, Nanjing, 210009, China; 3 State Key Laboratory of Natural Medicines, China Pharmaceutical University, Nanjing 210009, China; 4 Department of Organic Chemistry, School of Science, China Pharmaceutical University, Nanjing, 210009, China

## Abstract

Heat-shock protein 90 (Hsp90) is highly expressed in many tumor cells and is associated with the maintenance of malignant phenotypes. Targeting Hsp90 has had therapeutic success in both solid and hematological malignancies, which has inspired more studies to identify new Hsp90 inhibitors with improved clinical efficacy. Using a fragment-based approach and subsequent structural optimization guided by medicinal chemistry principles, we identified the novel compound **CPUY201112** as a potent Hsp90 inhibitor. It binds to the ATP-binding pocket of Hsp90 with a kinetic dissociation (K_d_) constant of 27 ± 2.3 nM. It also exhibits potent *in vitro* antiproliferative effects in a range of solid tumor cells. In MCF-7 cells with high Hsp90 expression, **CPUY201112** induces the degradation of Hsp90 client proteins including HER-2, Akt, and c-RAF. We prove that treating MCF-7 cells with **CPUY201112** results in cell cycle arrest and apoptosis through the wild-type (wt) p53 pathway. **CPUY201112** also synergizes with **Nutlin-3a** to induce cancer cell apoptosis. **CPUY201112** significantly inhibited the growth of MCF-7 xenografts in nude mice without apparent body weight loss. These results demonstrate that **CPUY201112** is a novel Hsp90 inhibitor with potential use in treating wild-type p53 related cancers.

Heat-shock proteins (HSPs) are a class of molecular chaperones with critical biological functions such as establishing proper protein conformations, preventing improper associations and collecting termed clients^1^. As a critical family member, Hsp90 mediates the maturation and stabilization of client proteins including kinases (HER-2, Akt, c-RAF and Cdk 4), receptors (androgen and estrogen receptor), and transcription factors (mutant p53, HIF-1α) in an ATP-dependent manner^2^,^3^,^4^. The maintenance of oncogenic client proteins requires high Hsp90 activity and consequently leads to the overexpression of Hsp90 in cancer cells. As a result, Hsp90 stands at the center of oncogenic proteostasis. Targeting Hsp90 through potent inhibitors provides a promising area of cancer chemotherapy^5^.

The natural products **Geldanamycin**^6^ and **radicicol**^7^ are early Hsp90 inhibitors, competing with ATP for the ATP-binding pocket of the Hsp90 N-terminal domain, blocking the folding of client proteins, and subsequently leading to their degradation through the ubiquitin-proteasome pathway. The **Geldanamycin** semi-synthetic derivatives 17-allylamino-17-demethoxy-geldanamycin (**17-AGG**) and 17-dimethylaminoethylamino-17-demethoxygeldanamycin (**17-DMAG**) are now in clinical trials^8^. However, they suffer from limitations including poor aqueous solubility, low bioavailability, potential multidrug efflux and hepatotoxicity^9^. To address these problems, a variety of inhibitors were recently discovered, including intravenous drugs such as **NVP-AUY922** (Novartis, phase II)^10^, **AT-13387** (Astex, phase II)^11^, **ganetispib** (STA-9090, Synta, phase II)^12^, **KW-2478** (Kyowa Hakko Kirin, phase I/II)^13^, **XL-888** (Exelixis, phase I)^14^, **PU-H71** (Memorial Sloan-Kettering Cancer Center, phase I)^15^, and **BIIB028** (Biogen Idec, phase I, structure undisclosed) and oral drugs including **DEBIO-0932/CUDC305** (Debiopharm, phase I)^16^, **MPC-3100** (Myrexis, phase I)^17^, **PF-4929113/SNX-5422** (Pfizer, phase I)^17^, **BIIB021** (Biogen Idec, phase II)^18^ and **NVP-Hsp990** (Novartis, phase I)^19^.

Here, we disclose the structure and activity of a novel Hsp90 inhibitor with a radicicol scaffold, **CPUY201112**. It was identified through shape-based virtual screening in our laboratory and later guided by fragment-based design. Rapid Overlay of Chemical Structures (ROCS) is a fast, shape-comparison application based on the idea that molecules have similar shapes if their volumes overlay well, and any volume mismatch is a measure of dissimilarity^20^,^21^. In a previous study, we performed shape-based similarity screening through ROCS overlays based on **CUDC-305**, **BIIB021**, **PU-H71** and **PU-3** and discovered a series of pyrazolopyrimidine analogs as HSP90 inhibitors^22^. The ligand-based method guided the researchers to efficiently identify novel inhibitors, especially for those targets with potent ligands.

In the present study, we selected the potent clinical compound **AT-13387** with a resorcinol core as the reference molecule for the ROCS model construction. By screening the Topscience database, we discovered 11 compounds containing a similar scaffold as the Hsp90 inhibitor. To improve the potency of these compounds, we designed and synthesized the analogs aided by structure-based design using docking simulation. **CPUY201112** was the most potent Hsp90 N-terminal inhibitor. Some of this work has been published^23^.The synthetic route and identification of **CPUY201112** is in supporting information (see Figs S1–3) In this study, we found that **CPUY201112** could bind to the ATP-binding pocket of sHp90 and disrupt its chaperone function. Cell biology studies showed that **CPUY201112** prevented the growth of a series of cancer cells by inducing apoptosis. *In vitro* studies showed that **CPUY201112** potently downregulated key client proteins such as HER-2, Akt, and c-RAF. The apoptosis-inducing effect of **CPUY201112** depended on a wild-type (wt) p53 signaling pathway. Accordingly, **CPUY201112** showed a synergistic effect with the MDM2 inhibitor **Nutlin-3a** in suppressing the proliferation of MCF-7 cells. Taken together, **CPUY201112** provides a novel and druggable Hsp90 inhibitor chemotype and is a promising compound that deserves further preclinical studies.

## Results

### CPUY201112 binds to the N-terminal ATP-binding site in Hsp90

**Figure 1:**
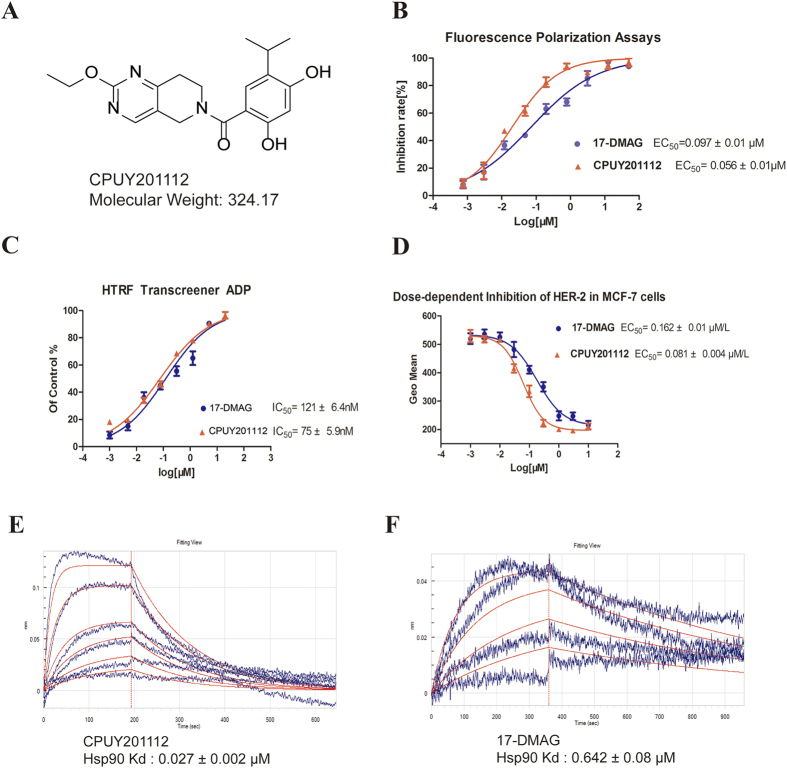
CPUY201112 binds to the N-terminal ATP-binding site in Hsp90. (**A**) The structure of CPUY201112. (**B**) Fluorescence polarization competition curves for **CPUY201112** and **17-DMAG**. (**C**) Inhibition of Hsp90 ATPase by **CPUY201112** and **17-DMAG** as detected by the HTRF transcreener ADP kit. Test compounds were diluted into a concentration series, incubated with Hsp90 and ATP, and the generated ADP was detected. (**D**) The EC_50_ curve of **CPUY201112** induced HER-2 degradation in MCF-7 cells. After treatment with **CPUY201112** and subsequent anti-HER-2 antibody staining, MCF-7 cells were collected and analyzed by a FACSCalibur flow cytometer. (**E**) and (**F**) The K_d_ of **CPUY201112** and of **17-DMAG**, respectively, with Hsp90. **17-DMAG** was chosen as positive control. All data were performed in triplicate and analyzed by GraphPad Prism.

**CPUY201112** is a novel, synthetic inhibitor of Hsp90 obtained from shape-based virtual screening and designed using a fragment-based approach in our laboratory. The synthesis route and H-NMR information are in the supplemental material. The structure of **CPUY201112** is shown in Fig. 1A. **CPUY201112** is a druggable small compound with a low molecular weight of 324.17.

To detect the direct binding of **CPUY201112** to the Hsp90 protein, we performed a real-time binding assay using the label-free biomolecular binding ForteBio Octet Red 96 system (Fig. 1E,F). **CPUY201112** directly bound to the Hsp90 protein with a kinetic dissociation constant (K_d_) of 0.027 ± 0.005 μmol/L, which was much more potent than **17-DMAG** (0.642 ± 0.08 μmol/L). **CPUY201112** bound to Hsp90 more stably and efficiently than did **17-DMAG**. A fluorescent polarization assay with FITC-labeled geldanamycin as probe was applied to evaluate the affinity between **CPUY201112** and Hsp90^24^. **17-DMAG**, a natural Hsp90 inhibitor, was chosen as a positive control. **CPUY201112** exhibited a similar binding manner as **17-DMAG**, competitively binding to the ATP pocket at the N-terminus of Hsp90. The IC_50_ values for **CPUY201112** and **17-DMAG** to Hsp90 were 0.056 ± 0.003 μmol/L and 0.097 ± 0.005 μmol/L, respectively (Fig. 1B). The high potency of **CPUY201112** towards Hsp90 prompted us to investigate its effect on the biological function of Hsp90. Hsp90 is an ATPase that can catalyze ATP hydrolysis without any substrate. The effects of different concentrations of **CPUY201112** on the ATPase activity of Hsp90 were evaluated by a homogeneous time resolved fluorescence (HTRF) Transcreener ADP assay. **CPUY201112** inhibited Hsp90 ATPase activity in a dose-dependent manner, with an equal inhibitory efficiency as **17-DMAG** (Fig. 1C). We further explored whether **CPUY201112** inhibited the ATPase activity of Hsp90 at the cellular level. HER-2 degradation is regarded as an on-target effect of Hsp90 inhibitors^25^. HER-2 expression levels were monitored in MCF-7 cells. Upon incubation with compounds for 24 h, HER-2 levels in MCF-7 cells were measured by flow cytometry. **CPUY201112** promoted the degradation of HER-2, with an EC_50_ of 0.081 ± 0.01 μmol/L (Fig. 1D). These experiments prove that **CPUY201112** binds to the N-terminal ATP-binding site in Hsp90 with high affinity, serving as a promising Hsp90 inhibitor.

**Figure 2:**
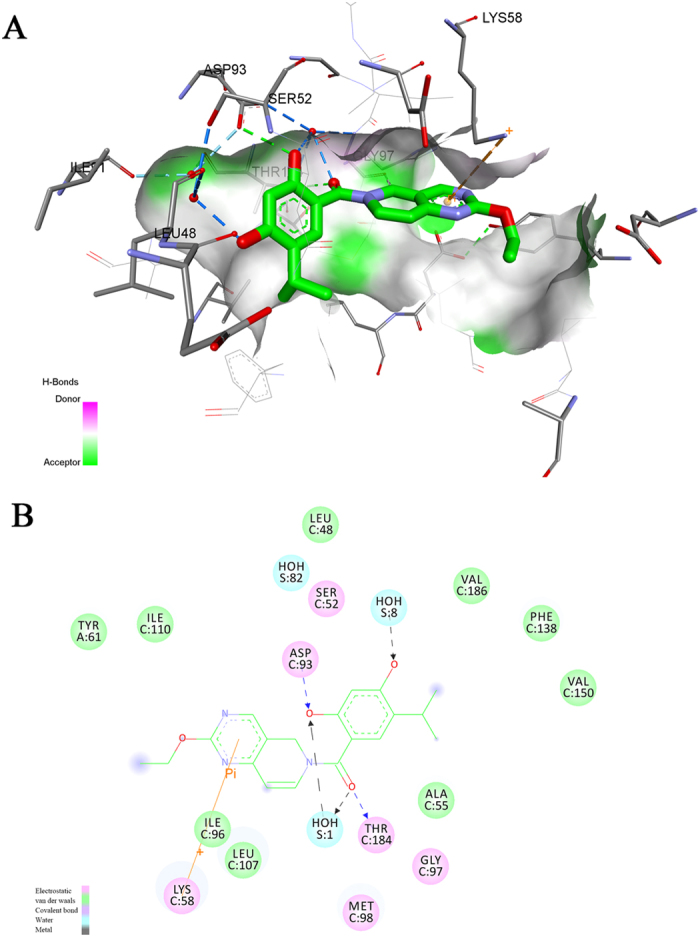
The binding mode between CPUY201112 and the ATP-binding site of Hsp90. (**A**) The 3D diagram of **CPUY201112** binding to Hsp90. **CPUY201112** is shown as a stick, with carbon atoms colored in green, oxygen atoms colored in red and nitrogen atoms colored in blue. The surface was added to the binding site of Hsp90, and the key residues in Hsp90 for binding were shown as sticks colored in grey. Intermolecular interactions were described using dot lines. (**B**) The 2D diagram of **CPUY201112** binding to Hsp90. Different intermolecular interactions are shown in various colors. The figure was prepared in Discovery Studio 4.0.

To further analyze the binding mode between **CPUY201112** and Hsp90, we docked **CPUY201112** to the ATP binding site of Hsp90 by GOLD binding software. The result (Fig. 2) showed that **CPUY201112** could insert into the binding groove of the ATP binding site of Hsp90 in a rational conformation. The resorcinol core pointed to the polar region of the site, comprised of Asp93, Ser52 and Thr184. The two hydroxyl groups on the resorcinol formed hydrogen bonds with Asp93 and Thr184 either directly or indirectly through conserved water molecules. The resulted hydrogen bond networks indicated a strong intermolecular interaction leading to the high binding affinity of **CPUY201112** to Hsp90. The carbonyl group linking the resorcinol and the tetrahydroisoquinoline core interacted with the side chain of Thr184 and a water molecule through hydrogen bonds. The tetrahydroisoquinoline core inserted into a hydrophobic pocket and formed multiple intermolecular interactions through van der Waals forces. Moreover, the tetrahydroisoquinoline core also formed a polar recognition with Lys58 through a sigma-pi interaction, which enhanced the binding affinity of **CPUY201112**. The ethyoxyl was imbedded into an open hydrophobic pocket formed by Ile96, Leu107 and Ile110. In summary, **CPUY201112** bound to Hsp90 in a reasonable manner, competing with ATP and blocking the biological function of Hsp90.

### CPUY201112 inhibited tumor cell proliferation

**Figure 3:**
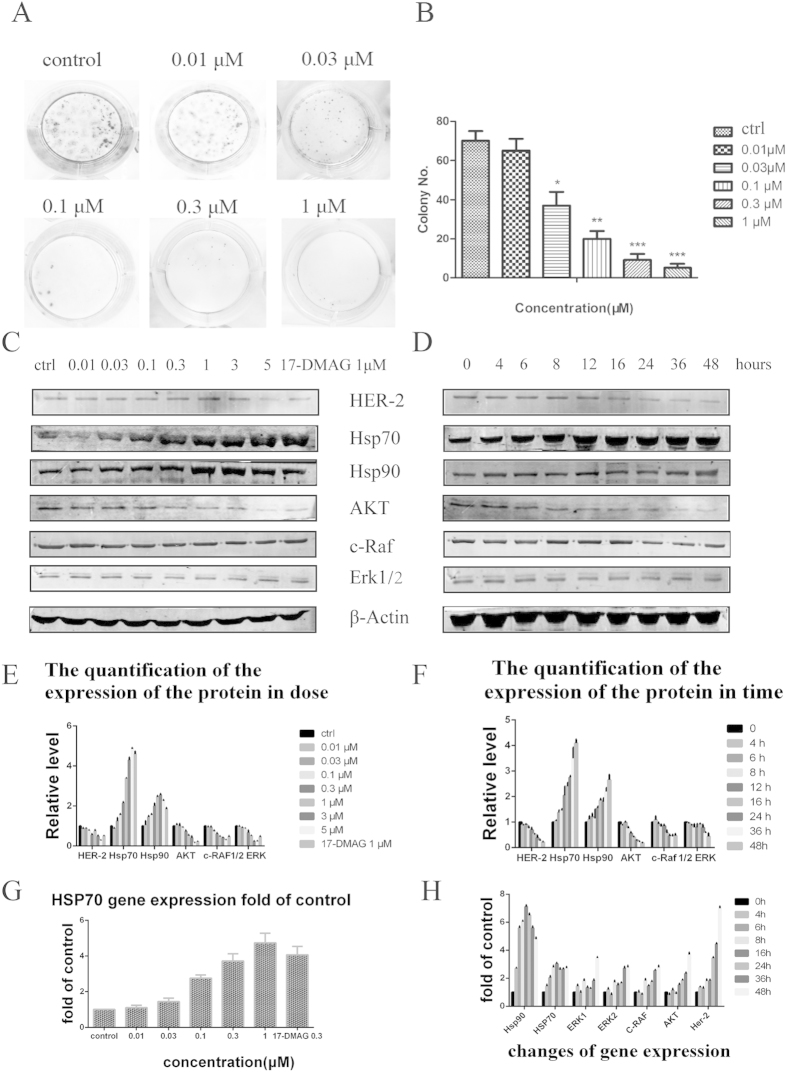
(**A**) Clonogenicity assay of MCF-7 cells in response to **CPUY201112**. (**B**) The colony quantity analysis. (**C**) Western blots of Hsp90 client proteins and heat shock proteins after treatment with indicated concentrations **CPUY201112** in MCF-7 cells for 24 h. (**D**) MCF-7 cells were treated with **CPUY201112** at a concentration of 1 μM for different times. Then, the cells were collected and prepared for western blot analysis. (**E,F**) The quantification of the protein expression. The blots are cropped and the gel were run under the same condition. **(G)** Induction of Hsp70 gene expression by **CPUY201112**. MCF-7 cells were transfected with a luciferase reporter whose expression was driven by the human Hsp70 promoter. After **CPUY201112** treatment at the indicated concentration for 12 h, the luciferase activity was assessed. All assays were repeated three times. *p < 0.05, **p < 0.01 and ***p < 0.001 versus control. (**H**) Changes in gene expression induced by **CPUY201112**. RT-PCR was used to detect the related gene expression as the change of delivery time.

The cell growth-inhibitory activity of **CPUY201112** was determined in cell lines with a range of **CPUY201112** concentrations for three days, followed by colorimetric measurement of cell viability using a [3-(4,5-dimethylthiazol-2-yl)-5-(3-carboxymethoxyphenyl)-2-(4-sulfophenyl)-2H-tetrazolium] inner salt assay. Consistent with its ability to inhibit HER-2 levels in cells, **CPUY201112** exhibited a broad spectrum of anti-proliferative activities. It decreased the viability of diverse cancer cell lines in a dose-dependent manner (Table 1), including HCT116 colon cells, HepG2 hepatocellular carcinomas, and other cancer cells. Breast cancer cell lines displayed the widest range of sensitivity to **CPUY201112**. Although **CPUY201112** had good water solubility and membrane permeability, **17-DMAG** had better activity in some cancer cells, including HT29 cells. However, **17-DMAG** belongs to the enzoquinone ansamycin compounds and will be metabolized to quinones, which are toxic to cells. Compared to the natural product with poor physicochemical properties, **CPUY201112** had good anti-proliferative activity in many types of cancer cells (Table 1). We also measured cell growth potentials using colony formation assays. After **CPUY201112** treatment for seven days, the number of surviving MCF-7 cell colonies dramatically decreased (Fig. 3A,B). **CPUY201112** at 1 μM completely inhibited colony formation. These results clearly confirmed that **CPUY201112** inhibited the growth of MCF-7 cells. To evaluate the safety of **CPUY201112**, we determined its toxicity in normal endothelial cells. Normal endothelial cells were less sensitive to **CPUY201112** (IC_50_, 7.01 ± 0.89 μM) than to **17-DMAG** (IC_50_, 0.239 ± 0.04 μM), indicating that **CPUY201112** might preferably inhibit cancer cell growth and thus have a better therapeutic index.

### Effects of CPUY201112 on Hsp90 client proteins

Hsp90 regulates the correct conformation and activity of many client proteins and plays key roles in tumor survival and progression^26^. Therefore, Hsp90 inhibition could reduce client protein expression. We next examined the effect of **CPUY201112** on the expression and/or phosphorylation of Hsp90 client proteins by western blotting. A range of **CPUY201112** concentrations were tested for 24 h in MCF-7 cells that overexpressed HER-2. As shown in Fig. 3C, treatment significantly downregulated the level of the client proteins c-RAF, Akt and 1/2Erk in a dose-dependent manner. As expected, the expression levels of Hsp70 and Hsp90 were enhanced dose-dependently. In a time course assay, the same group of client proteins and heat shock proteins were again analyzed by western blotting. MCF-7 cells were treated with 1 μmol/L **CPUY201112** for different time periods up to 48 h. As shown in Fig. 3D, MCF-7 cells treated with **CPUY201112** exhibited reduced HER-2 levels. Akt and c-RAF degradation was observed from 6 h after treatment. The decreased Her-2, Akt, c-Raf and Erk1/2 protein levels were not due to the inhibition of gene transcription, as their mRNA levels were not altered and were even enhanced by **CPUY201112**, as measured by RT-PCR (Fig. 3H). Again, the increased Hsp70 level was detected from 6 h and persisted for up to 48 h after treatment. These results were consistent with the Hsp70 luciferase reporter assay (Fig. 3G). **CPUY201112** activated the Hsp70 promoter in a dose-dependent manner, which is a compensatory response to Hsp90 inhibition. These results suggest that **CPUY201112** eliminated pro-proliferative and pro-survival signaling by destabilizing c-RAF/Akt through inhibiting Hsp90 chaperone activity.

### CPUY201112 induces MCF-7 cell cycle arrest and apoptosis

**Figure 4:**
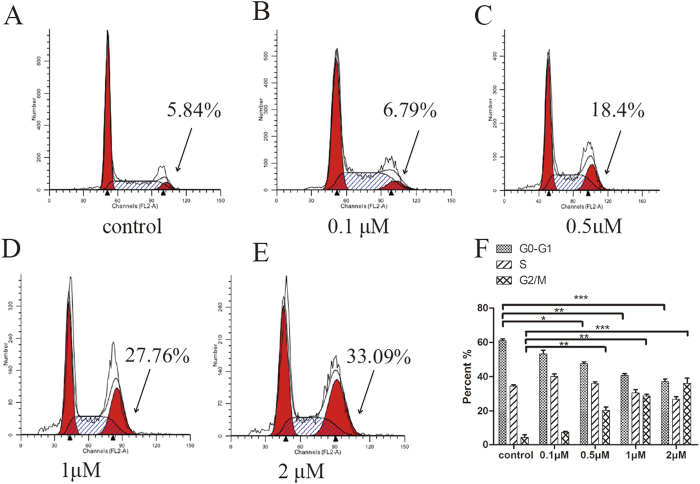
CPUY201112 induces MCF-7 G2/M cell-cycle arrest and apoptosis. (**A**) Control. (**B**) 0.1 μM. (**C**) 0.5 μM. (**D**) 1 μM. (**E**) 2 μM. (**F)** The cell cycle percentages were quantified using Flow Cytometry. *p < 0.05; **p < 0.01. MCF-7 cells were treated with **CPUY201112** at the indicated concentrations. Cells were harvested, stained with propidium iodide and analyzed by flow cytometry to determine DNA content.

**Figure 5:**
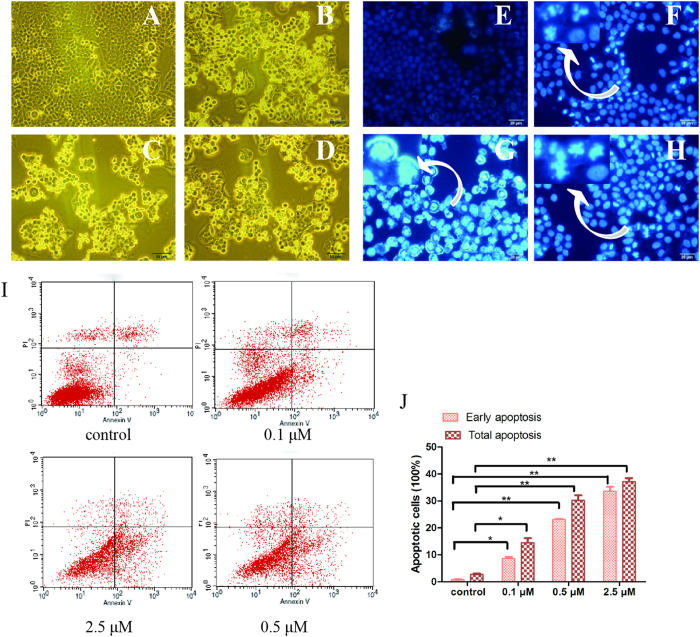
CPUY201112 induces MCF-7 cell apoptosis. (**A–D**) Morphological changes of cancer cells and nuclei. (**E–H**) MCF-7 cell apoptosis was induced by **CPUY201112**. Cultured MCF-7 cells were treated with **CPUY201112** at the indicated concentrations for 48 h. The cells were then were stained with DAPI and observed by fluorescence microscopy. (**A,E**) Control. (**B,F**) 0.1 μM. (**C,G**) 0.5 μM. (**D, G**) 2.5 μM. (**I**) **CPUY201112** induces apoptosis in a dose-dependent manner. MCF-7 cells were treated with different concentrations of **CPUY201112** for 48 h, and Annexin V and propidium iodide staining was performed. (**J)** The early apoptosis and total apoptosis percentages were quantified using flow cytometry. *p < 0.05; **p < 0.01.

Hsp90 inhibition in cancer cells would induce apoptosis and cell cycle arrest^27^. Cell cycle analysis was performed to determine whether **CPUY201112** induced the accumulation of MCF-7 cells in the G2/M stage. As shown in Fig. 4, **CPUY201112** remarkably induced the accumulation of MCF-7 in the G2-M phase within 24 h, with a concomitant loss of G1-G0 phase. However, the S phase changed little, suggesting that CPUY201112 induced MCF-7 cell cycle arrest at the G2/M phase. Over this period, the percentage of apoptotic cells increased. When the cells were treated for 48 h, the apoptosis-inducing effect became more apparent. We next evaluated the influence of **CPUY201112** on the cell skeleton using morphological observation. Incubation of **CPUY201112** with MCF-7 cells resulted in phenotypic changes such as distortion, membrane blebbing and shrinkage. A large proportion of cells became round in shape and showed necrosis. In comparison, cells in the untreated group grew well with clear cytoskeletons (Fig. 5A–D). As identified by DAPI staining, the bright nuclear condensation and the apoptotic bodies appeared after treatment with **CPUY201112**, whereas the untreated cells displayed normal shapes and clear skeletons (Fig. 5E–H). In MCF-7 cell lines, **CPUY201112** resulted in the enhanced, dose-dependent induction of apoptosis compared with the control. Treatments with 2.5 μM of **CPUY201112** for 48 h induced more than 35% of cells into apoptosis (Fig. 5I). These results indicate that **CPUY201112** induces anti-proliferative activity through apoptosis mediated by cell cycle arrest and client protein degradation.

### CPUY201112 inhibits tumor growth in animals

**Figure 6:**
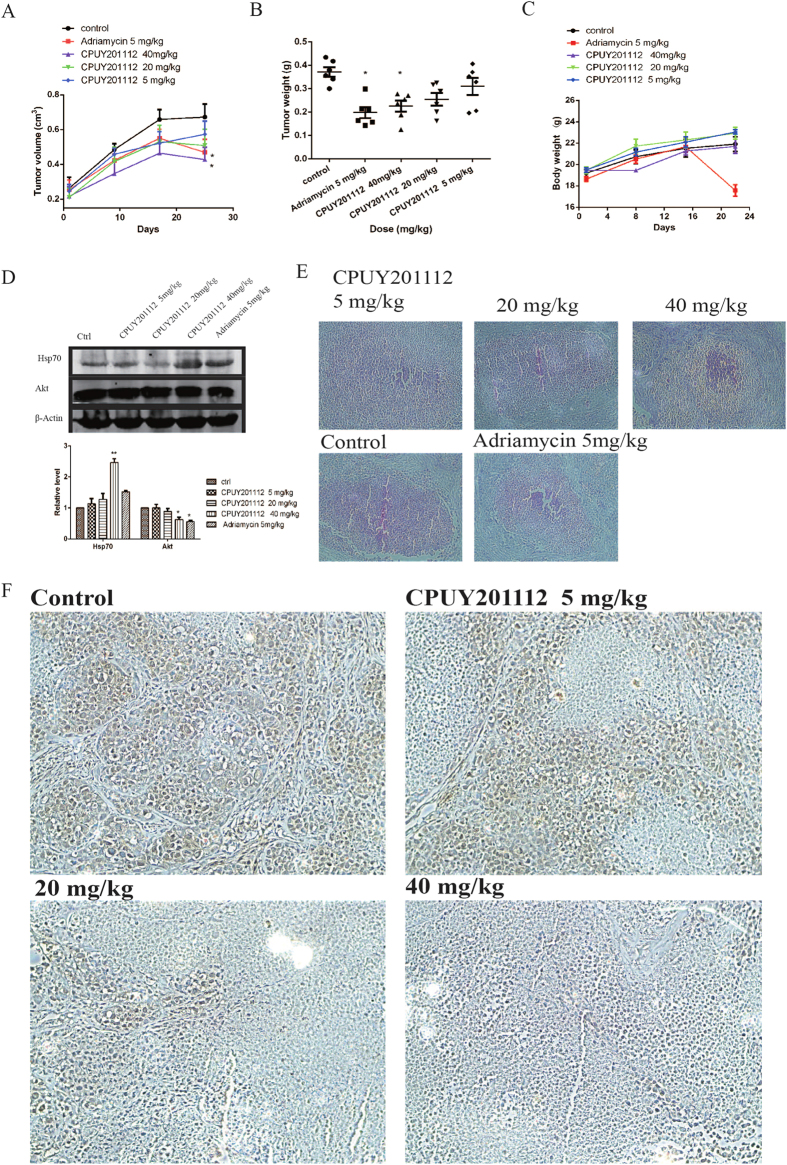
CPUY201112 inhibits the growth of MCF-7 xenografts in nude mice. (**A–C**) Female nude mice implanted with MCF-7 xenografts were treated with 5 mg/kg or 10 mg/kg of **CPUY201112** for 24 days. Tumor diameters were measured and used to calculate tumor volumes (**A**). After 24 days, the mice were sacrificed, and individual tumors were weighed (**B**). *p < 0.05, student *t*-test (n = 6). (**D**) Western blot analysis of Hsp70 and Akt in the tumor. *p < 0.05; **p < 0.01; Student *t*-test (n = 3). The blots are cropped and the gel were run under the same condition. (**E**) Representative H&E images of individual tumors. (**F**) Immunohistochemical evaluation to assess HER-2 levels in MCF-7 tumor tissue after treatment with **CPUY201112**.

To determine whether the *in vitro* effects of **CPUY201112** translate to antitumor efficacy *in vivo*, **CPUY201112** activity was evaluated at multiple doses in MCF-7 tumor xenograft models. After tumors were established, we treated mice with 5, 20, or 40 mg/kg of **CPUY201112** through intraperitoneal injection every four days for 24 days. Consistent with its *in vitro* anti-tumor activity, **CPUY201112** inhibited tumor growth, resulting in significantly reduced tumor volumes and weight (Fig. 6A–C). Adriamycin, a classic chemotherapeutic drug in breast cancer, was chosen as positive control. Treatment with 5 mg/kg, 20 mg/kg and 40 mg/kg of **CPUY201112** decreased the tumor volume by 11.92%, 26.58% and 39.63%, respectively, compared with the control group. At the same time, we measured the tumor size using hematoxylin-eosin staining. As shown in Fig. 6E, the tumor cells had a decreasing trend with increasing doses of **CPUY2011112**. Although 5 mg/kg Adriamycin contributed to the 42.63% inhibition ratio, there was a significant reduction in the weight of nude mice. Half of the Adriamycin group was dead at the last day of dosing, and the other half were in poor health. In contrast, the nude mice in the **CPUY201112** group had normal weights and quality of life, suggesting that **CPUY201112** had reduced side effects. This result was consistent with the feature that **CPUY201112** preferably targeted tumor cells. At the same time, the Hsp90 co-chaperone protein Hsp70 and the client protein Akt were detected in tumor tissue (Fig. 6D). Treatment with 40 mg/kg **CPUY201112** remarkably induced the expression of Hsp70 and decreased the expression of Akt, suggesting that tumor regression was caused by **CPUY201112** through targeting Hsp90. In a further experiment, formalin-fixed, paraffin-embedded MCF-7 tumor sections were prepared for immunohistochemical staining for the HER-2 protein. HER-2 levels decreased markedly on the surface of tumor cells with increasing dose. (Fig. 6F). Taken together, we demonstrated that **CPUY201112** inhibited tumor growth *in vivo*.

### CPUY201112 enhances *wt*-p53 signaling and kills cancer cells in a *wt*-p53-dependent manner

**Figure 7:**
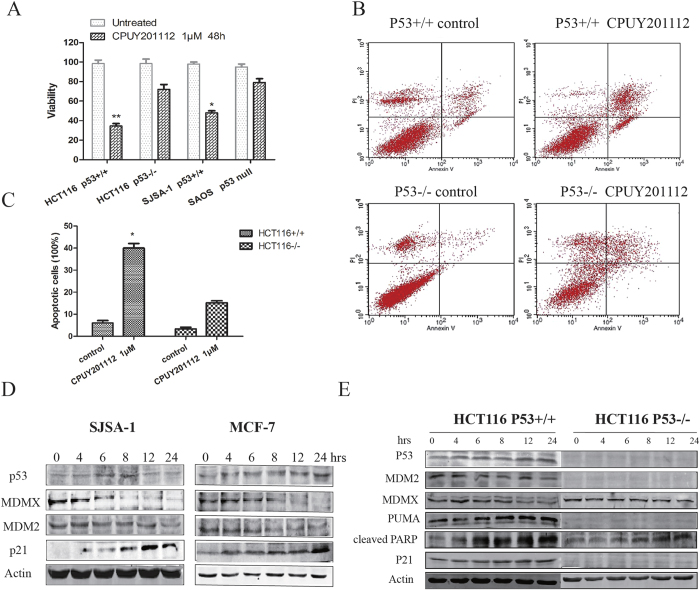
CPUY201112 enhances *wt*-p53 signaling and kills cancer cells in a *wt*-p53-dependent manner. (**A**) Cell viability assay. Cell lines with different p53 states were treated with **CPUY201112** for 48 h, and the viability of cells was detected by the MTT method. (**B**) **CPUY201112** shows better cytotoxic cell killing in the *wt*-p53 tumor cells HCT116 p53^+/+^ versus HCT116 p53 ^−/−^, as demonstrated by Annexin-V and PI staining and measured by FACS. (**C**) The quantification of cell apoptosis induced by 2 μM **CPUY201112** in HCT116 p53^+/+^ and HCT116 p53^−/−^ after treatment for 24 h. (**D**) **CPUY201112** increases p53 and p21 but decreases MDM2 and MDMX protein levels. MCF-7 and SJSA-1 human cancer cells were treated with 2 μM of **CPUY201112** for the indicated time periods. The levels of p53, MDMX, MDM2 and P21 proteins were analyzed by immunoblots. Actin was used as a loading control. The blots are cropped and the gel were run under the same condition. (**E**) In response to **CPUY201112**, p53^−/−^ tumor cells show much lower PARP cleavage and lack p21 and PUMA induction compared with isogenic p53^+/+^ cells. The cell pairs were treated with 2 μM **CPUY201112** or DMSO. The levels of the indicated proteins were analyzed by immunoblotting.

P53 is an important client protein of the Hsp90 chaperone machinery^28^. Inhibition of Hsp90 by **17-AGG** or **17-DMAG** increased the expression of *wt*-p53 and promoted p53-dependent apoptosis in both mouse embryo fibroblasts and in allotransplanted primary medulloblastomas *in vivo*^27^,^29^. Therefore, we examined whether **CPUY201112** also affected *wt*-p53 signaling. We first chose human cancer cell lines from solid tumors with high *wt*-p53 expression, including the human colon cancer cell line HCT116 and the human osteosarcoma cell line SJSA-1. **CPUY201112** showed preferential cytotoxicity in *wt*-p53 cancer cells as measured by cell viability assays (Fig. 7A). In contrast, the HCT116 p53^−/−^ cell line or the p53 null human tumor cell line SAOS had weaker cytotoxic responses to **CPUY201112**. To further study whether **CPUY201112** induced apoptosis in a *wt*-p53-dependent manner, the isogenic colorectal cancer line pair HCT116 p53^+/+^ and p53^−/−^ were exposed to **CPUY201112**. In Annexin-V/PI analysis, **CPUY201112** induced more apoptosis in the HCT116 p53^+/+^ cells compared with the HCT116 p53^−/−^ cells (Fig. 7B,C). To monitor apoptosis in this pair of cell lines, we also detected the caspase activation level. Consistent with the results of the Annexin-V/PI assay, caspase activation in HCT116 p53^+/+^ cells was higher than that in p53^−/−^ cells, suggesting that the apoptosis induced by **CPUY201112** depended on p53 status in cells (Supplemental Fig. 4). Cleaved PARP is an important marker of apoptosis, and PUMA protein is a critical mediator of p53-induced apoptosis. Therefore, we measured the levels of these proteins in these cells. PUMA expression was induced in HCT116 p53^+/+^. In contrast, no obvious change was detected in HCT116 p53^−/−^ cells (Fig. 7E). The cleaved PARP was also induced in HCT116 p53^−/−^ cells, suggesting that other pathways may contribute to apoptosis, which requires further investigation. However, from these results we concluded that **CPUY201112** induced p53-mediated apoptosis in HCT116 cells.

At the same time, p53 pathway marker proteins such as MDM2, MDMX and p21 were also detected after **CPUY201112** treatment for 24 h in the *wt*-p53 cells, and the results are shown in Fig. 7D,E. **CPUY201112** increased the expression of p53 and its partner p21 in a time-dependent manner in the cell lines. At the same time, **CPUY201112** decreased the MDMX and MDM2 protein levels. MDM2 is an Hsp90 client that is decreased after Hsp90 inhibition^30^. We performed Quantitative real-time polymerase chain reaction (qRT-PCR) to detect the transcriptional regulation of the p53 signaling pathway (Supplemental Figs S4 and 5). **CPUY201112** increased the p53 mRNA level. Simultaneously, **CPUY201112** also enhanced the expression of the conventional p53 targets p21, MDM2, and PUMA, confirming p53 activation. However, MDMX mRNA levels were not affected by **CPUY201112** treatment, suggesting that the down-regulation of MDMX protein may occur at the posttranscriptional level. In conclusion, **CPUY201112** enhanced *wt*-p53 signaling and killed cancer cells in a *wt*-p53-dependent manner.

### CPUY201112 synergized with Nutlin-3a to induce apoptosis

**Figure 8:**
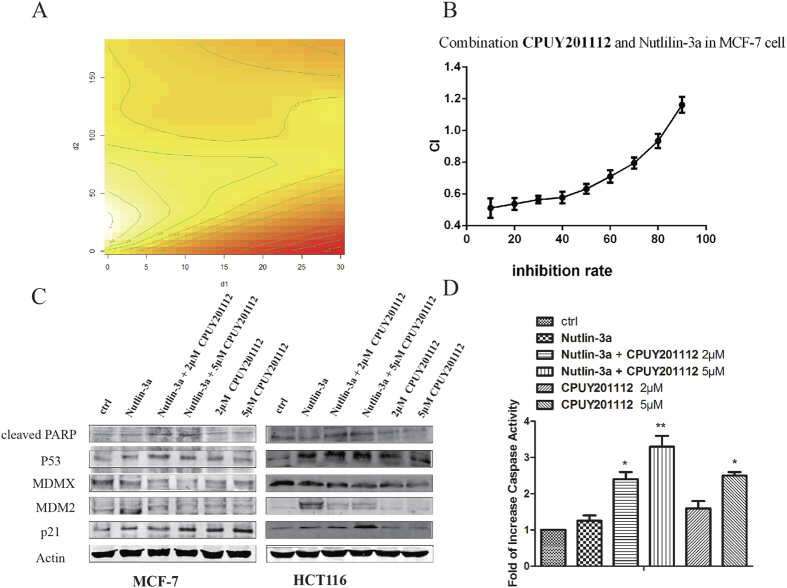
CPUY201112 synergizes with Nutlin-3a to induce apoptosis. (**A**) The contour map of the combined effect of **CPUY201112** and **Nutlin-3a**. d_1_ and d_2_ represent the concentration of **CPUY201112** and **Nutlin-3a**, respectively. In this contour map, f (d1, d2) >0 indicates a synergistic effect, and f (d1, d2) <0 indicates antagonism. (**B**) Synergistic induction of cell death by **CPUY201112** and **Nutlin-3a**. MCF-7 cell were treated with increasing doses of **CPUY201112** or **Nutlin-3a** alone or in combinations at a fixed ratio for 24 h. The % rate of dead cells was determined by MTT assays. CIs were calculated by Chou-Talalay combination index analysis. (**C**) **CPUY201112** increases p53 and p21 and decreases MDM2 levels in **Nutlin**-treated cells. Cells were pretreated with 2 or 5 μM of **CPUY201112** for 2 h followed by 10 μM of **Nutlin-3a** for an additional 24 h where indicated. Treatment with **CPUY201112** alone was for 24 h. Actin was a loading control. The blots are cropped and the gel were run under the same condition. (**D**) Increased caspase activity upon combined treatment. MCF-7 cells were treated as shown, and caspase activity was measured. The error bars represent standard errors from three replicates.

**Nutlin-3a**, a well-known MDM2 inhibitor, could inhibit MDM2 protein activity, leading to higher *wt*-p53 levels and enhanced cancer cell death. A combination of **Nutlin-3a** and **CPUY201112** may induce significantly more death than either alone. Next, we determined whether **CPUY201112** could synergize with MDM2 inhibitors in a cell viability assay. **CPUY201112** and **Nutlin-3a** were combined in the synergy assay by using the Chou-Talalay combination index analysis with the median effect principle^31^. To better show the effect of the combination of the two compounds, we also built a semi-parametric model to study the proportion with different doses. By utilizing the R software package GAM mgcv, a semiparametric model was estimated. We obtained the estimated values at different dose combinations^32^. In this contour map, f (d_1_, d_2_) >0 indicates a synergistic effect. If f (d_1_, d_2_) <0, that indicates antagonism. As illustrated in Fig. 8A, combinations of the two compounds produced improved cell killing effects, and there was a synergistic effect in different dose proportions. The synergistic effect increased along with the increased dose proportion of **CPUY201112**. The combination index (CI) theorem of Chou-Talalay offers a quantitative definition for additive effect (CI = 1), synergism (CI < 1), and antagonism (CI > 1) in drug combinations. In our study, the combined effect of **CPUY201112** and **Nutlin-3a** on MCF-7 cell viability was assessed by the MTT assay, in which cells were treated with a serial dilution of **CPUY201112**/**Nutlin-3a** (1:1) mix to determine the combination index (CI_50_). As shown in Fig. 8B, the combination of the two drugs produced an enhanced anti-proliferative response with CI_50_ values less than 1 when the inhibition rate was below eight percent. CI_50_ < 1 is indicative of synergistic cell killing by a drug combination. Taken together, the combination of **CPUY201112** with **Nutilin-3a** resulted in enhanced induction of apoptosis compared with each single agent alone, which suggests a synergistic interaction between the compounds.

The **CPUY201112** and **Nutlin-3a** combinations also induced higher caspase levels in MCF-7 cells (Fig. 8D). PARP cleavage was also up-regulated with the combination (Fig. 8C). **Nutlin-3a** induces high expression of MDM2 but will not completely disrupt all p53-MDM2 complexes. Interestingly, **Nutlin-3a** and **CPUY201112** significantly down-regulated MDM2 protein levels compared with **Nutlin-3a** alone. Thus, **CPUY201112** enhanced **Nutlin-3a**’s efficacy by relieving MDM2 repression of p53 as well. As a result, p53 and p21 protein was also significantly up-regulated in MCF-7 and HCT116 cells upon combined treatment compared with **Nutlin-3a** or **CPUY201112** alone (Fig. 8C). In conclusion, **CPUY201112** enhanced the p53 signaling pathway to promote apoptosis.

## Discussion

In this article, we proved that **CPUY201112** is a novel Hsp90 inhibitor and that its molecular events lead to the anti-tumor activity.

Hsp90 is highly expressed and plays a key role in cellular growth, differentiation, and survival^33^. In tumor development, Hsp90 is crucial for the stability and function of many oncogenic proteins, including kinases, transcription factors, and hormone receptors^4^,^34^. Hsp90 inhibition is a promising therapeutic approach in the clinic, especially in advanced stages of clinical development of specific molecular-defined subgroups of cancers (e.g., ALK-rearranged non-small-cell lung cancer, multiple myeloma and HER-2 amplified breast cancer)^1^,^35^,^36^. Therefore, finding novel, small-molecule inhibitors that are effective against Hsp90 is a promising strategy for new cancer treatments. Although many structurally diverse Hsp90 inhibitors have been developed in the past decades, more efforts are being made to improve the therapeutic index of Hsp90 inhibitors, including chemical optimization to increase affinity or selectivity and decrease toxicity and optimization of drug dosing and scheduling^37^,^38^. In this study, we designed a new, synthetic and potent compound **CPUY201112** as an Hsp90 inhibitor. Structurally, **CPUY201112** is a resorcinol derivative that belongs to the new generation of Hsp90 inhibitors with improved safety and pharmacokinetic properties. **CPUY201112** has a unique scaffold that is considerably smaller than some second generation Hsp90 inhibitors such as **BIIB021** and **AT-13387**. In the target-binding assay, **CPUY201112** competed with geldanamycin to bind to the ATP-binding site of Hsp90. In the binding affinity assay, **CPUY201112** showed higher binding affinity for Hsp90 than **17-DMAG**. Hsp90 as an ATPase exerts a chaperone role through an ATP-driven conformational change. **CPUY201112** with its smaller volume may have entered the ATP-binding pocket in the so-called closed and open conformations. In contrast, due to its larger size, **17-DMAG** only occupies the ATP-binding pocket in the open conformation. This explained why **CPUY201112** performed better than **17-DMAG** in the Hsp90 ATPase assays.

Because a large number of the oncoproteins are known Hsp90 clients, including EGFR, c-MET, BCR-ABL, B-RAF, c-KIT and HER-2, inhibiting Hsp90 in cells can lead to client degradation via the proteasome pathway^39^. The panel of breast cancer lines is typically characterized by high HER-2 expression that contributes to proliferation and survival, so they are sensitive to Hsp90 inhibitors. A cell-based HER-2 degradation assay was applied to evaluate the Hsp90 inhibitor^40^. **CPUY201112** induced the degradation of HER-2 with an EC_50_ value of 81 nM in MCF-7 cells. Other key client proteins such as AKT, EKR1/2, and c-RAF were degraded in the cell-based assay. **CPUY201112** treatment in MCF-7 cells resulted in the degradation of key client proteins and the compensatory increase of Hsp90 and Hsp70 in a time- and dose-dependent manner. **CPUY201112** caused AKT/c-RAF to be degraded, which resulted in the inhibition of AKT/c-RAF-mediated signaling and eventual tumor cell apoptosis. **CPUY201112** simultaneously influenced multiple signaling pathways and may be more effective in solving the drug resistance problem. At the same time, **CPUY201112** inhibited the growth of many human tumor cell lines *in vitro*. Compared with **17-DMAG**, **CPUY201112** inhibited the growth of normal endothelial cells and cancer cells with a larger gap. The IC_50_ value for endothelial cells was approximately 10-fold higher than that for MCF-7 cells. These results suggested that **CPUY201112** provided a relatively wide therapeutic window for breast cancer. **CPUY201112** significantly inhibited the growth of MCF-7 xenografts in nude mice without apparent body weight loss.

**CPUY201112** treatment rapidly and dramatically reduced proliferation and induced apoptosis. The treatment of MCF-7 cells with **CPUY201112** for 24 h induced noticeable accumulation in the G2/M phase with a concomitant loss of G1-G0 phase. Simultaneously, Akt and c-RAF, and Erk1/2 inhibition also contributed to MCF-7 cell apoptosis. Accordingly, AKT pathway activation promoted cell survival by phosphorylating and inactivating pro-apoptotic proteins^41^. However, the marked apoptotic induction was observed after **CPUY201112** treatment for 48 h. The apoptosis induced by **CPUY201112** correlated with the irreversible commitment to growth arrest and the effects on the cell cycle.

P53 is an important tumor suppressor gene, suppressing tumor cell proliferation and preventing tumor development^42^. Wild-type p53 plays a central role in 50% of human tumors and can induce tumor cell apoptosis. Therefore, the strategies for targeted reactivation of *wt*-p53 have high clinical value. Indeed, many clinical anti-cancer drugs induce apoptosis through *wt*-p53 activation^43^,^44^,^45^. The Hsp90 inhibitor **17-DMAG** induces cell death with a requirement for *wt*-p53^27^. In our work, we showed that **CPUY201112** enhanced *wt*-p53 signaling and killed cancer cells in a *wt*-p53-dependent manner. A panel of human cell lines treated with **CPUY201112** showed different sensitivities, suggesting that p53 was involved in the apoptosis induced by **CPUY201112**. Indeed, Hsp90 inhibitors directly or indirectly affect the p53 signaling pathway by modulating the client proteins. As the results show, the decreased Akt protein expression induced by **CPUY201112** would prevent phosphorylation and maintain the MDM2 levels, contributing to the accumulation of *wt*-p53. **Nutlin-3a** is a potent and highly selective compound that can cut off the interaction between p53 and MDM2, releasing p53 and resulting in cancer cell death^46^. However, in clinical trials **Nutlin-3a** suffers from problems with inefficiently inducing definitive apoptosis, especially in most solid tumors^47^. Several recent studies showed that simultaneous inhibition of PI3K/AKT signaling would increase apoptosis in ALL or CLL leukemia, and suppression of Erk signaling synergistically improved **Nutlin**-induced apoptosis in AML^46^. Our study found that **CPUY201112** efficiently synergized with **Nutlin-3a** to induce apoptosis in a p53-dependent manner. The major effect of **CPUY201112** was inducing definitive apoptosis by modulating p53 signaling, whereas **Nutlin-3a** induced transient cell cycle arrest. Therefore, **CPUY201112** could be a compelling partner for **Nutlin-3a** in a broad spectrum *wt*-p53-harboring solid tumors.

The activation of heat shock transcription factor HSF1 by Hsp90 N-terminal inhibitors has been reported in both preclinical studies and clinical evaluations^48^. HSF1 induces the transcription of Hsp70, Hsp27 and to some degree Hsp90 itself to protect cancer cells from apoptosis^49^. This drawback of Hsp90 N-terminal inhibitors contributes to drug resistance and other side effects in clinical trials. Unfortunately, in our study **CPUY201112**, an N-terminal inhibitor, also induced the transcription and expression of Hsp70 and Hsp90. Silencing of either Hsp70 or HSF1 dramatically increased cancer cell sensitivity to Hsp90 inhibition and induction of apoptosis^50^. However, more efforts are needed to avoid the heat shock stress induced by Hsp90 N-terminal inhibitors. Several approaches are now being investigated to enhance cancer cell sensitivity to Hsp90 inhibitors, including targeting Hsp70, HSF1, Hsp27, and the C-terminus of Hsp90^2^,^51^,^52^. Fortunately, some compounds have been reported to inhibit Hsp90 without inducing heat shock stress^53^. In our laboratory, Hsp90 C-terminal inhibitors are currently being studied.

In summary, we have developed and characterized a new small-molecule Hsp90 inhibitor with potent antitumor effects both *in vitro* and *in vivo*. **CPUY201112** demonstrated potent pharmacologic properties. Importantly, **CPUY201112** synergized with **Nutlin-3a** against *wt*-p53-harboring solid tumors. Therefore, **CPUY201112** represents a new chemical entity as an Hsp90 inhibitor for the cancer treatment.

## Materials and Methods

### Compounds and reagents

**CPUY201112** (prepared in our laboratory) and **17-DMAG** (Selleck, TX, USA) were dissolved in DMSO (Sigma, St. Louis, MO, USA) and stored in aliquots at −20 °C for no more than one month before use. The vehicle (DMSO) was used as a control in experiments at a maximum concentration of 0.1%. The system for the detection of immunoblotted proteins was from LI-COR (Odyssey, NE, USA). All other chemicals and biochemistry reagents were obtained from Sigma-Aldrich (St Louis, MO, USA). The following antibodies were used at appropriate concentrations as recommended by the manufacturer: anti-poly (ADP-ribose) polymerase (PARP) (# 9542), anti-AKT (# 9272s), anti-c-RAF (# 9422), anti-phospho AKT (# 9275s), anti-Hsp90 (# 4874), anti-Hsp70 (# 4876), anti-Erk (# 4370), anti-p21 (# 2947), and anti-HER-2/ErbB2 (# 2242) were purchased from Cell Signaling Technology (Beverly, MA, USA). Anti-p53 (OP43) and anti-MDM2 (07–575) were purchased from Merck Millipore (Billerica, MA, USA). Anti-MDMX (ab16058) was from Abcam (Cambridge, MA, USA). β-actin antibodies was purchased from LSBio (Seattle, WA, USA). The HTRF transcreener ADP kit was from Cisbio Bioassays (BP, Codolet, France).

### Preparation of Hsp90

The cloning, expression and purification of recombinant human Hsp90α have been described previously^54^. The region encoding full-length Hsp90 was subcloned into pET28a. Protein expression in *E. coli* cells was induced with 0.5 mM IPTG. Cells were harvested after 20 h of growth at 16 °C and then disrupted by sonication. The soluble lysate was clarified by centrifugation and applied to a Ni^2+^-nitrilo-triacetic acid (NTA) agarose column (QIAGEN) in buffer (50 mM Tris-Cl, 300 mM NaCl, 10 mM imidazole, 10% [v/v] glycerol, 10 mM PMSF, 10 mM DTT). Hsp90 protein was eluted with a linear gradient of 20–1,000 mM imidazole. Hsp90 was identified by SDS-PAGE, and the highly concentrated fraction was dialyzed against buffer (20 mM Tris-Cl, pH 7.5; 6 mM MgCl_2_; 20 mM KCl) and then aliquoted, frozen in liquid nitrogen, and stored at −80 °C.

### Cell Culture

The human cancer cell lines MCF-7, Sk-br-3, MDA-MB-231, BT-474, U2OS, A549, HT29, HepG2, SGC7901, and PC-3 were purchased from American Type Culture Collection (ATCC) or Cell Culture Center at the Institute of Basic Medical Sciences, Chinese Academy of Medical Sciences. HCT116 with p53^−/−^ and p53^+/+^, SAOS, SJSA-1, and U2OS cells were kindly gifted by professor Wang Shaomeng at the University of Michigan. A549 cells were cultured in F12K supplemented with 10% fetal calf serum and antibiotics. Hep-G2 and MCF-7 cells were cultured in MEM. HCT116, SAOS, and HT29 cells were cultured in McCOY’s 5A, and other cancer cell lines were cultured in supplemented RPMI-1640 medium. Cells were maintained in a 37 °C incubator (Thermo, USA) with 95% humidity and 5% CO_2_.

### Animals

Female nude mice were supplied by the Shanghai Slac Laboratory Animal Limited Company. This experiment was conducted in accordance with the Animal Ethics Committees of the Institute of Materia Medica, Chinese Academy of Medical Sciences and China Pharmaceutical University. Female BALB/c nude mice (5–6 weeks old) with body masses ranging from 18 to 22 g were purchased from the Shanghai Institute of Materia Medica, Chinese Academy of Sciences. Animals were maintained in a pathogen-free environment (23 ± 2 °C and 55 ± 5% humidity) on a 12 h light ∼12 h dark cycle with food and water supplied ad libitum throughout the experimental period. The animals were housed and cared for in accordance with the guidelines established by the National Science Council of Republic of China.

### Cell proliferation and colony formation assay

Cell viabilities were determined by a colorimetric assay using 3-(4, 5-dimethylthiaz-ol-2-yl)-2, 5- diphenyltetrazoliumbromide (MTT, Sigma, St. Louis, MO) as described previously^54^. Cells that were given only culture media were used as control. After incubation for 72 h, absorbance (A) was measured at 570 nm. For colony formation assays, cells (100 cells/plate) were plated in 6-well plates. The day after seeding, cells were treated with inhibitors for 14 days. The media were changed every four days. When colonies started appearing on day 21, visible cells were washed twice with ice cold PBS and fixed with 10% ice cold methanol for 10 min and then stained with 0.5% crystal violet (Sigma, St. Louis, MO, USA) in 25% ice cold methanol for an additional 10 min. Following many washes with double distilled water, the plates were dried at room temperature and images were scanned using a scanner.

### Fluorescence polarization competition assay and ForteBio Octet RED 96 System assay

Hsp90α (100 nmol/L) and FITC-geldanamycin (10 nmol/L) were incubated in a 96-well microplate at room temperature in the presence of assay buffer containing 20 mmol/L HEPES (pH 7.4), 50 mmol/L KCl, 5 mmol/L MgCl_2_, 20 mmol/L Na_2_MoO_4_, 2 mmol/L dithiothreitol, 0.1 mg/mL bovine gamma globulin, and 0.1% (v/v) 3 [(3-cholamidopropyl)-dimethylammonio]-1-propane sulfonate (90 μL). After 1 h equilibration in the dark, compounds were added as 10-fold stocks in 10 μL of a 20% DMSO/80% assay mix. The reaction was incubated for 3 h at room temperature. The fluorescence was then measured in an Analyst plate reader (Molecular Devices SpectraMax Paradigm; Ex 485 nM, Em 535 nM). Wells containing no compound or no Hsp90 were used as high or blank controls, respectively. The IC_50_ values were calculated using a four-parameter curve in GraphPad Prism 5.0. The binding kinetic constant of **CPUY201112** to Hsp90 was determined by ForteBio Octet RED 96 system. First, the Hsp90 protein was incubated with biotin. **CPUY201112** and **17-DMAG** were diluted into different concentrations. Then, the Kd values were calculated by the SSA sensor (Fortebio).

### Homogeneous time resolved fluorescence (HTRF) Transcreener ADP assay

The HTRF transcreener ADP kit was purchased from Cisbio Bioassays. For the inhibition experiment, Hsp90, ATP and compounds were prepared in the enzymatic buffer provided with the kit and supplemented with 10 mM MgCl_2_. Hsp90 protein at 200 nM was first pre-incubated at 37 °C for 1.5 h in the presence of various concentrations of compound. Then, ATP (100 μM) and HTRF detection reagents were added, and the plate was incubated at room temperature for 30 min. The fluorescence was then measured in an Analyst plate reader (Molecular Devices SpectraMax Paradigm; Ex 620 nM, Em 665 nM). The IC_50_ was calculated by a four-parameter curve using GraphPad Prism.

### HER-2 Degradation Assay

The assay was performed as described previously^40^. MCF-7 cells (5 × 10^5^ per well) were plated in 24-well plates for 24 h. Then, cells were incubated in the presence of serially diluted compounds in RPMI-1640. After 24 h, cells were rinsed with PBS and then trypsinized. Following centrifugation, the cell suspensions were washed in PBS with 0.2% bovine serum albumin and 0.2% sodium azide (defined as BA buffer) and then resuspended in 100 μL anti-HER-2 IgG antibody (eBioscience). Cells were incubated 30 min at room temperature, washed twice in 200 μL BA buffer, resuspended in 400 μL BA buffer, and transferred to 5 mL round polystyrene tubes. Samples were analyzed using a FACSCalibur FlowJo cytometer.

### Hsp70 luciferase reporter assay

The HspA1A promoter-driven luciferase plasmid was gifted by Professor Jianhua Liu. The experimental method followed their protocol with a little modification^55^. Briefly, MCF-7 cells were grown in 24-well plates. The cells were co-transfected with pHspA1A-Luc and pRL-SV40 (a plasmid encoding Renilla luciferase) using Lipofectamine 2000. After 6 h, the cells were treated with the indicated concentrations of the various compounds for 24 h. Luciferase activity was assessed by the dual-luciferase reporter assay system (Promega, E1910) using a luminometer (Thermo Scientific Luminoskan Ascent). The level of Hsp70 activation for a sample was calculated as the ratio between firefly and Renilla luciferase activity for the same sample. Typically, each sample was run in triplicate. Results were obtained from at least three separate experiments.

### Cell death and cell cycle analyses

To detect the morphological evidence of apoptosis, MCF-7 cell nuclei were visualized following DNA staining with the fluorescent dye DAPI (Sigma-Aldrich, St. Louis, MO, USA). The methods have been described previously^54^.

After treatment with **CPUY201112**, cells were trypsinized and diluted to 1 × 10^6^ cells/mL followed by 15 min of incubation with Annexin V and PI staining (BD Biosciences) in the dark at room temperature. The cell suspensions were adjusted to 500 μL with binding buffer and counted using a FacScan analyzer (argon laser; Becton Dickinson, USA). All data were analyzed using Flow software. To analyze the intracellular DNA content, cells were treated with serum-free culture medium for 24 h and then with the compound for another 24 h. Cells were harvested and fixed in 70% ethanol at 4 °C overnight. Cells were washed with PBS, suspended in PBS containing 50 mg/mL propidium iodide (PI) (Sigma–Aldrich, St. Louis, MO, USA) and 100 mg/mL RNase A, incubated at 37 °C for 0.5 h and analyzed with FACScaliber. Data output was analyzed using Cell Quest software (Becton Dickinson). All experiments were conducted in triplicate.

For cell death assays, caspase assays were performed in triplicate using the fluorimetric Homogeneous Caspases Assay (Roche). Briefly, 4 × 10^4^ cells plated per well in a 96-well plate were treated with 100 μL of media containing different concentrations of compound. The substrate working solution was added, and the plate was incubated at 37 °C for 6 h or at room temperature overnight in the dark. The plates were read by Thermo Scientific Varioskan Flash with an excitation wavelength of 485 nm and emission wavelength of 520 nm.

### A statement identifying the institutional and/or licensing committee experimental approval

All animal experiments were handled according to the Animal Ethics Committees of the Institute of Materia Medica, Chinese Academy of Medical Sciences. The animal protocol was approved by and carried out according to the Institutional Animal Care and use Committee of China Pharmaceutical University.

### Human tumor xenograft studies

Female nude mice were injected subcutaneously with 1 × 10^7^ MCF-7 breast cancer cells at the dorsa. Four weeks later, tumors were cut into 3 mm × 3 mm × 3 mm pieces and implanted subcutaneously in the dorsa of female nude mice with a trocar. Animals implanted with xenografts were randomized into five groups one week later, when tumors reached an average volume of 100 mm^3^. Mice were injected peritoneally with 5, 20, or 40 mg/kg of CPUY201112 or vehicle in a volume of 10 ml/kg daily for three weeks. Mice were monitored daily for signs of toxicity, and tumor diameters were measured three times per week with a caliper. Tumor volumes were calculated using the formula: volume = (width)^2^ × length/2. Mice were sacrificed three weeks later, and tumors were weighed and fixed in 10% formaldehyde for histological examinations.

### Histology and immunohistochemical staining

Tumors were embedded in paraffin for sectioning. For histological examinations, five micron serious sections were stained by H&E. Immunohistochemical staining was carried out as described previously^21^. Briefly, sections were de-paraffinized and rehydrated followed by antigen retrieval using hot citrate buffer. Sections were then blocked in 5% normal horse serum and 1% normal goat serum and incubated with the Her-2 antibody (1:400, Abcam, Cambridge, MA, USA) for 2 h. The DAB Kit (Vector, Burlingame, CA, USA) was used to visualize the staining according to the manufacturer’s instructions.

### Molecular docking

Compound **CPUY201112** was first imported to Discovery Studio (DS) 3.0 and then ionized. The 3D conformation was generated by the ‘Prepare Ligands’ protocol in DS at pH 7.0. The Powell conjugate gradient algorithm was then applied for the minimization of the hit compounds (convergence criterion of 0.0005 kcal/mol/M, energy minimizations in 20,000 steps) in CHARMm forcefield. The molecular docking was carried out using GOLD 5.1 software, considered one of the most successful and widely used docking programs. In the present study, the Hsp90-ligand complex (PDB id: 2XJX) was downloaded from the Protein Data Bank (PDB) for docking studies. Residues around the original ligand (radius 7.5 Å) that completely covered the ATP binding site were defined as the binding site. Docking studies were performed using the standard default settings with 100 GA runs of molecules. For each GA run, a maximum of 125,000 operations was performed. Considering ligand flexibility, special care was taken to include options such as flipping of ring corners, pyramidal nitrogens, amides, secondary and tertiary amines, and rotation of carboxylate groups as well as torsion angle distribution and post-process rotatable bonds as default. The annealing parameters were used as default. Hydrophobic fitting points were calculated to facilitate the correct starting orientation of the compound for docking by placing the hydrophobic atoms appropriately in the corresponding areas of the active site. Cutoff values of 3.0 Å for hydrogen bonds and 4.0 Å for van der Waals interactions were set. The docking was terminated when the top ten solutions attained root-mean-square deviation (RMSD) values within 1.5 Å.

### Statistical analysis

Results for continuous variables were presented as the mean ± standard error. Two-group differences in continuous variables were assessed by the unpaired T-test. P-values are two-tailed with confidence intervals of 95%. Statistical analysis was performed by comparing treated samples with untreated controls. All statistical tests were conducted using Prism software version 5.0 (GraphPad, San Diego, CA, USA).

## Additional Information

**How to cite this article**: Xu, X.-L. *et al*. CPUY201112, a novel synthetic small-molecule compound and inhibitor of heat shock protein Hsp90, induces p53-mediated apoptosis in MCF-7 cells. *Sci. Rep*. **6**, 19004; doi: 10.1038/srep19004 (2016).

## Supplementary Material

Supplementary Information

## Figures and Tables

**Table 1:** Antiproliferation activity of CPUY201112 in tumor cell lines and human umbilical vein endothelial cells in culture.

Cell lines	Origin	Growth inhibition (IC_50_)	
		CPUY201112 (μM/L)	17-DMAG (μM/L)
MCF-7	Breast carcinoma	0.624 ± 0.05	0.112 ± 0.08
Sk-Br-3	Breast carcinoma	0.429 ± 0.03	0.067 ± 0.06
MD-MBA-231	Breast carcinoma	2.32 ± 0.13	0.192 ± 0.11
BT-474	Breast carcinoma	0.384 ± 0.07	0.092 ± 0.03
HCT116	Colon carcinoma	0.763 ± 0.09	0.151 ± 0.04
HT29	Colon carcinoma	0.921 ± 0.1	0.089 ± 0.03
A549	Human pulmonary carcinoma	0.543 ± 0.05	0.109 ± 0.04
SGC-7901	Gastric cancer	0.498 ± 0.04	0.103 ± 0.02
U2OS	Human osteosarcoma	0.449 ± 0.08	0.192 ± 0.01
PC-3	Human prostate carcinoma	0.659 ± 0.05	0. 094 ± 0.02
HepG2	Hepatocellular carcinoma	0.342 ± 0.07	0. 092 ± 0.03
HUVECs	Human umbilical vein endothelial cells	7.01 ± 0.89 μM	0.239 ± 0.04
L-02	liver cells	8.23 ± 0.59	0.58 ± 0.08
HEK293	Human embryonic kidney 293 cells	4.19 ± 0.25	0.19 ± 0.08

^a^Values represent means ± standard deviations for at least two separate experiments performed in triplicate.
