# Supplementary material for: CPUY201112, a novel synthetic small-molecule compound and inhibitor of heat shock protein Hsp90, induces p53-mediated apoptosis in MCF-7 cells
Source: Sci Rep. 2016 Jan 8;6:19004. doi: 10.1038/srep19004 (PMC4705544; doi:10.1038/srep19004)
Supplement: Supplementary Information [file srep19004-s1.pdf]

CPUY201112, a novel synthetic small-molecule compound and inhibitor of heat shock protein Hsp90, induces p53-mediated apoptosis in MCF-7 cells

Xiao-Li Xu, [a,b] Qi-chao Bao, [a,b] Jian-Min Jia, [a,b] Fang Liu, [a,b] Xiao-Ke Guo, [a,b] Ming-ye Zhang, [a,b] Jin-lian Wei, [a,b] Meng-chen Lu, [a,b] Li-li Xu, [a,b] Xiao-Jin Zhang, [a,b,d] Qi-Dong You, [a,b,c] * and Hao-Peng Sun, [a,b,c]*

[a] Jiangsu Key Laboratory of Drug Design and Optimization, China Pharmaceutical University, Nanjing, 210009, China

[b] Department of Medicinal Chemistry, School of Pharmacy, China Pharmaceutical University, Nanjing, 210009, China

[c] State Key Laboratory of Natural Medicines, China Pharmaceutical University, Nanjing 210009, China

[d] Department of Org anic Chemistry, School of Science, China Pharmaceutical University, Nanjing, 210009, China

Hao-peng Sun: Fax & Tel: +86-25-83271216, E-mail: sunhaopeng@163.com;

Qi-dong You: Fax & Tel: +86-25-83271351, E-mail: youqidong@gmail.com.

1、The synthetic route of CPUY201112

Figure S1: Reaction Condition
Figure S2 : CPUY201112: ^{1}H NMR, in DMSO-d_6, 300 MHz
Figure S3 :CPUY201112: ^{13}C NMR, in DMSO- d_6, 75 MHz

2、The method and result of Real-time qRT-PCR

Figure S4: The caspase activity induced by the isogenic colorectal cancer pair HCT116 p53$^{+/+}$ and p53$^{-/-}$ cells.
Figure S5: CPUY201112 induces the transcription of p53 and its target genes p21, MDM2 in p53 $^{+/+}$.

3、The full-length blots are for key data

Supplement Figure S6: The blot is from Fig 3C in paper.
Supplement Figure S7: The blot is from Fig 3D in paper.
Supplement Figure S8: The blot is from Fig 3D in paper.
Supplement Figure S9: The blot is from Fig 3D in paper.
Supplement Figure S10: The blot is from Fig 3D in paper.

Supplement Figure S11: The blot is from Fig 6D in paper.

1、 The synthetic route of CPUY201112.

CPUY201112 was obtained from **36** (1.5 g, 3.00 mmol) and ethanol (solvent) in the general way. A white solid: yield 0.87 g (81 %); m.p. = 281.5 °C; ^{1}H NMR (300 MHz, *d6*-DMSO) δ 9.52-9.53 (m, 2H, Ar-OH), 8.42 (s, 1H, Ar-H), 6.89 (s, 1H, Ar-H), 6.38 (s, 1H, Ar-H), 4.49-4.58 (m, 2H, -NCH_2-), 4.29-4.31 (s, 2H, -OCH_2-), 3.68 (s, 2H, -CH_2N-), 3.06-3.16 (m, 1H, -CH-), 2.73-2.81 (m, 2H, -CH_2CH_2N-), 1.21-1.30 (m, 3H, -OCH_2CH_3), 1.09-1.11 (m, 6H, -CH_3); ^{13}C NMR (75 MHz, $CDCl_3$, δ ppm): 169.15, 165.43, 163.12, 157.11, 156.44, 152.76, 125.97, 125.50, 120.51, 113.53, 102.14, 62.40, 43.04, 42.22, 31.71, 25.79, 22.56 (2C), 14.27. IR(KBr):3299, 2960, 2864, 1601, 1436, 1350, 1259, 1219, 1109, 973, 927, 844, 789 cm^{-1}; HRMS (ESI): calcd for $C_{19}H_{23}N_3O_4$ $[M + H]^+$ 358.4036, found 358.4052. Purity: 98.37 % by HPLC (MeOH/H_2O = 80:20).

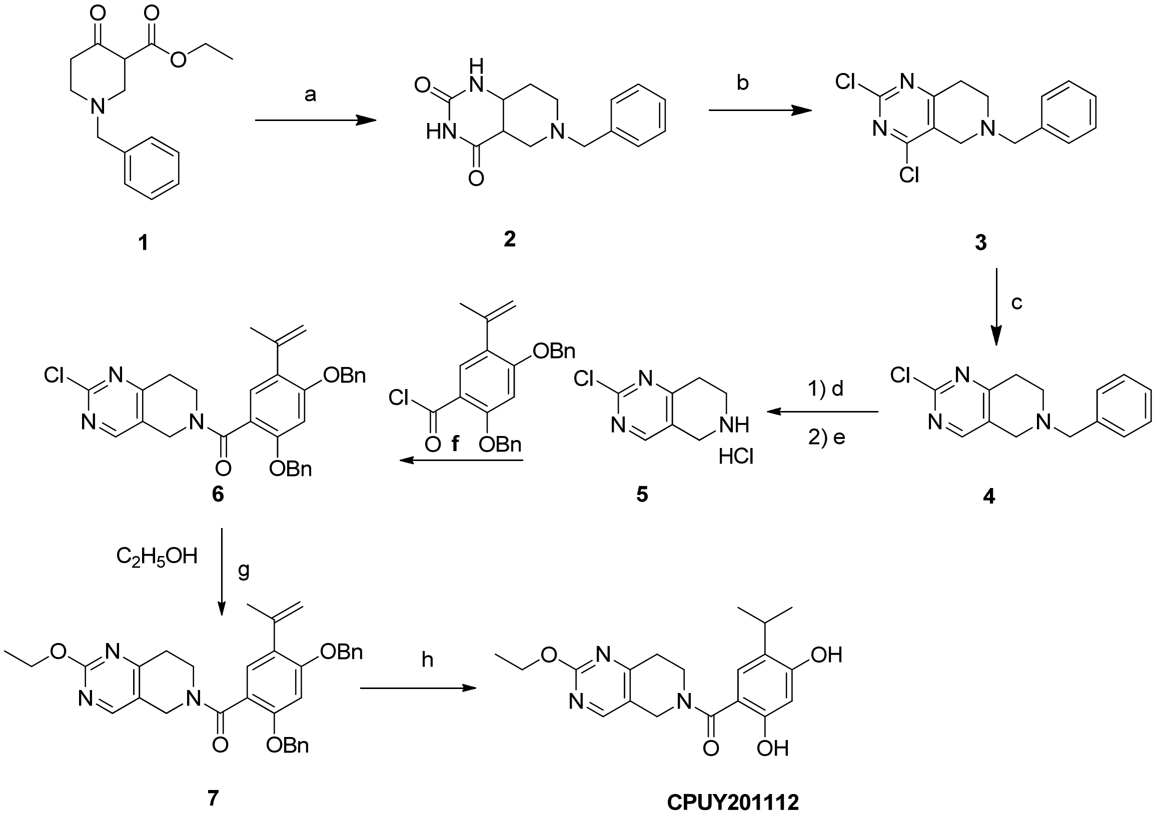

Supplement Figure S1: Reaction Condition: (a) urea, MeONa, EtOH, reflux, 24 h; (b) $POCl_3$, N_2, 3 h; (c) Activated Zn, NH_4OH, EtOH, reflux, 12 h; (d) ACE-Cl, CH_2Cl_2, 0 °C ~ room temperature,

reflux; (e) MeOH,reflux (f) EDCI, HOBt, Et_3N,CH_2Cl_2, room temperature, 12 h; (g) Na_2CO_3, 1,4-Dioxane, 85 ºC, 6 h; (h) 10% Pd/C, H_2, MeOH,room temperature, 15 h. Yield, 74.43%。

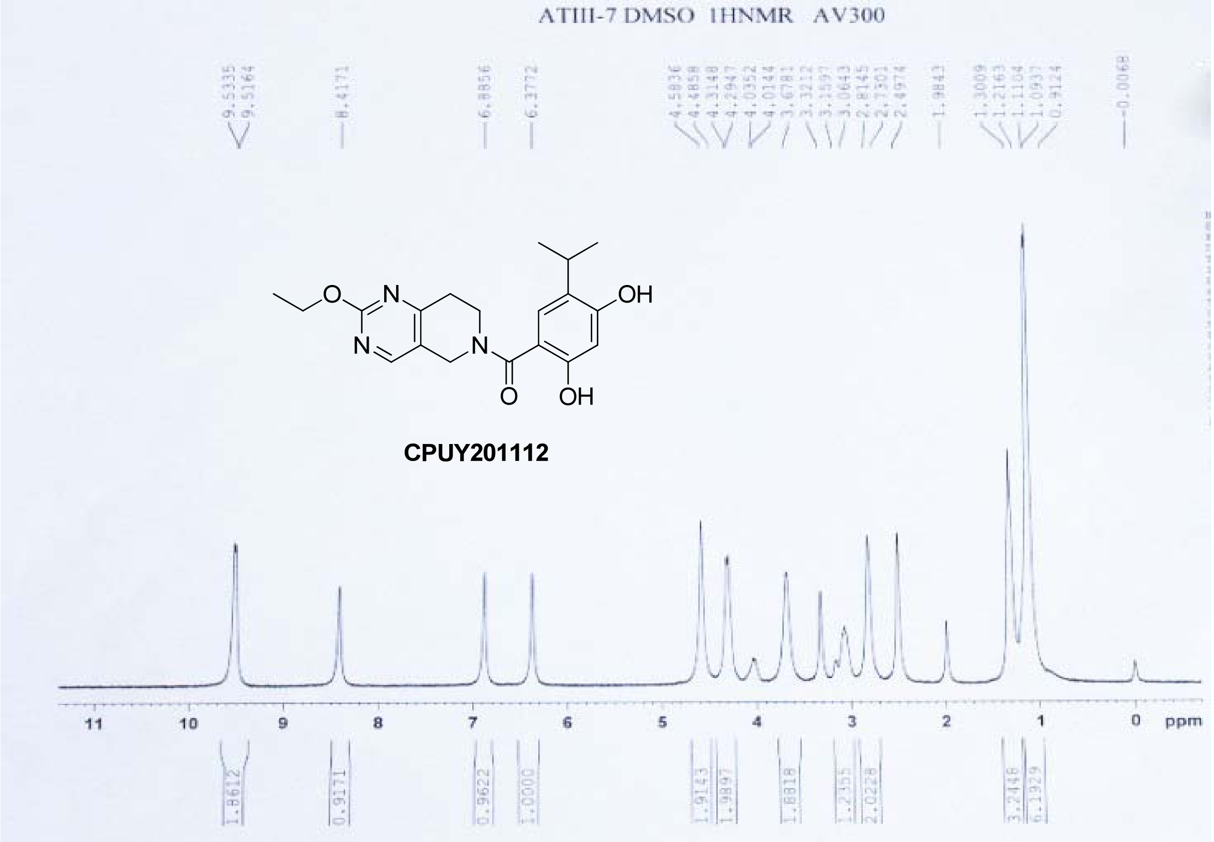

Supplement Figure S2 :CPUY201112: ^{1}H NMR, in DMSO-d_6, 300 MHz

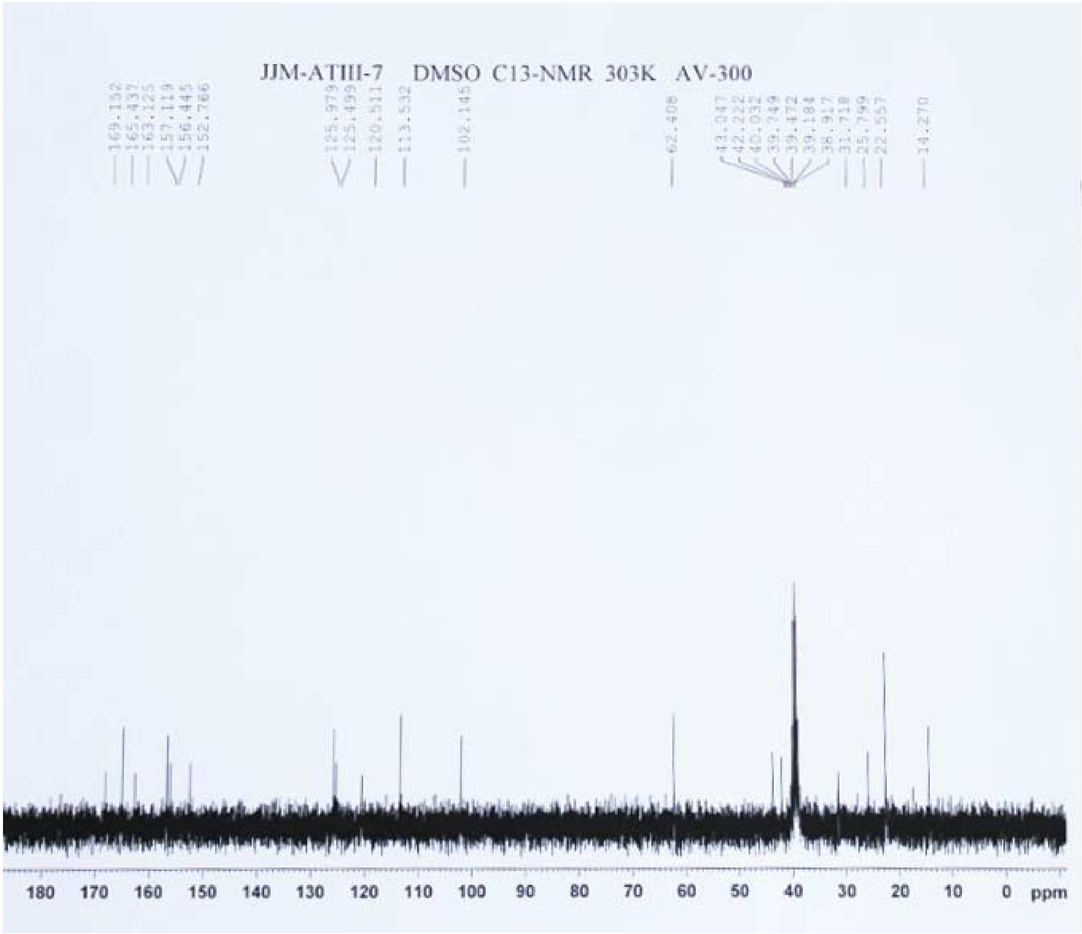

Supplement Figure S3 :CPUY201112: ^{13}C NMR, in DMSO-d_6, 75 MHz

2、 The method of Real-time qRT-PCR

Total RNA was isolated using TRIzol (Invitrogen) and after reverse transcription, real-time PCR was performed using a 7500 Fast Real time PCR system (Applied Biosystems). Details of primers used are described in SI Primers are shown as follows: MDM2, forward primer5’-GGCGATTGGAGGGTAGACCT-3’, reverse primer 5’-CACATTTGCCTGGATCAGCA -3’; MDMX forward primer 5’-GCCTTGAGGAAGGATTGGTA-3’, reverse primer 5’-TCGACAATCAGGGACATCAT-3’; P21, forward primer 5’-CTGGAGACTCTCAGGGTCGAAA-3’, reverse primer 5’-GATTAGGGCTTCCTCTTGGAGAA-3’; p53, forward primer 5’- CCA TGG AGG AGT CAC AGT CG -3’,reverse primer 5’- GCA GAG GCA GTC AGT CTG AGT C -3’; GAPDH (internal control), forward primer 5’-ACCACAGTCCATGCCATCAC-3’,reverse primer 5’-TCCACCACCCTGTTGCTGTA -3’.

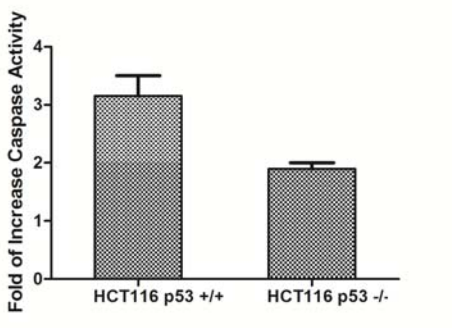

Supplement Figure S4 : The caspase activity induced by the isogenic colorectal cancer pair HCT116 p53$^{+/+}$ and p53$^{-/-}$ cells. Cells were treated with 2 μM of **CPUY201112** for 48 h and caspase activity was measured. The error bars represent standard errors from three replicates.

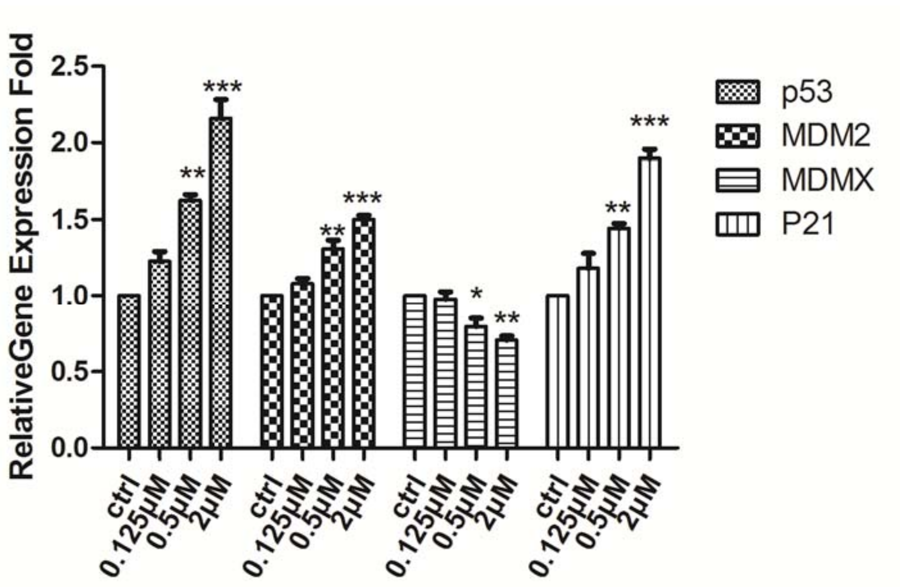

Supplement Figure S5: CPUY201112 induces the transcription of p53 and its target genes p21, MDM2 in p53 $^{+/+}$. HCT116 p53 $^{+/+}$ cell was treated with 1 μM of **CPUY201112** for 24h. mRNA levels were measured by qRT-PCR and normalized to GAPDH. The error bars represent standard errors from two independent experiments, each preformed in triplicated.

3、 The full-length blots are for key data

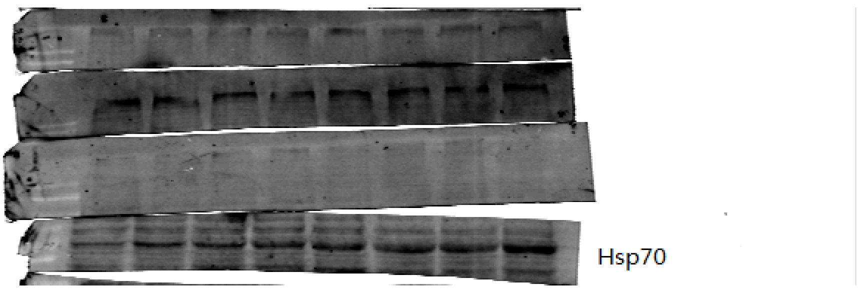

Supplement Figure S6: The blot is from Fig 3C in paper. Western blots of Hsp90 client proteins and heat shock proteins after treatment with indicated concentrations **CPUY201112** in MCF-7 cells for 24 h.

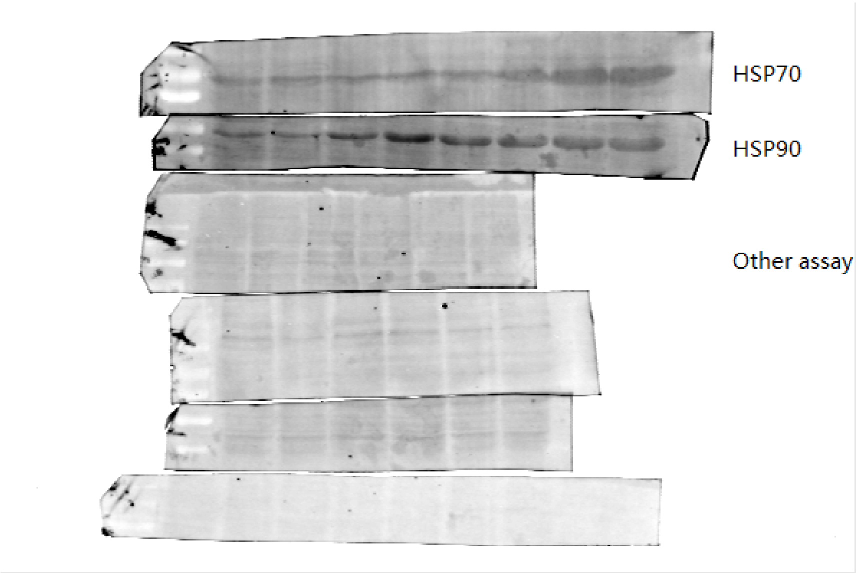

Supplement Figure S7: The blot is from Fig 3D in paper. MCF-7 cells were treated with **CPUY201112** at a concentration of 1 μM for different times. Then, the cells were collected and prepared for western blot analysis.

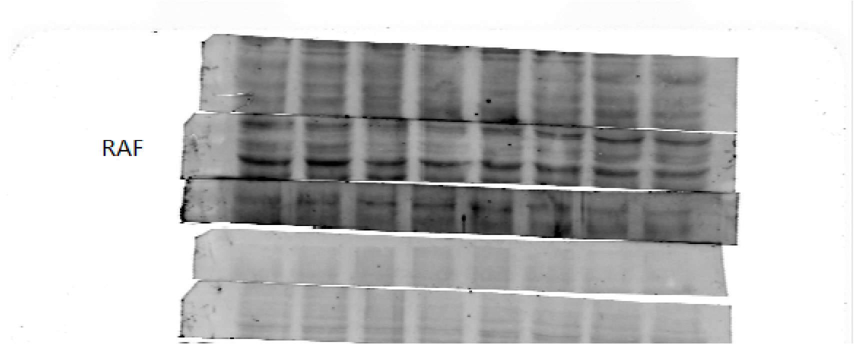

Supplement Figure S8: The blot is from Fig 3D in paper. MCF-7 cells were treated with **CPUY201112** at a concentration of 1 μM for different times. Then, the cells were collected and prepared for western blot analysis.

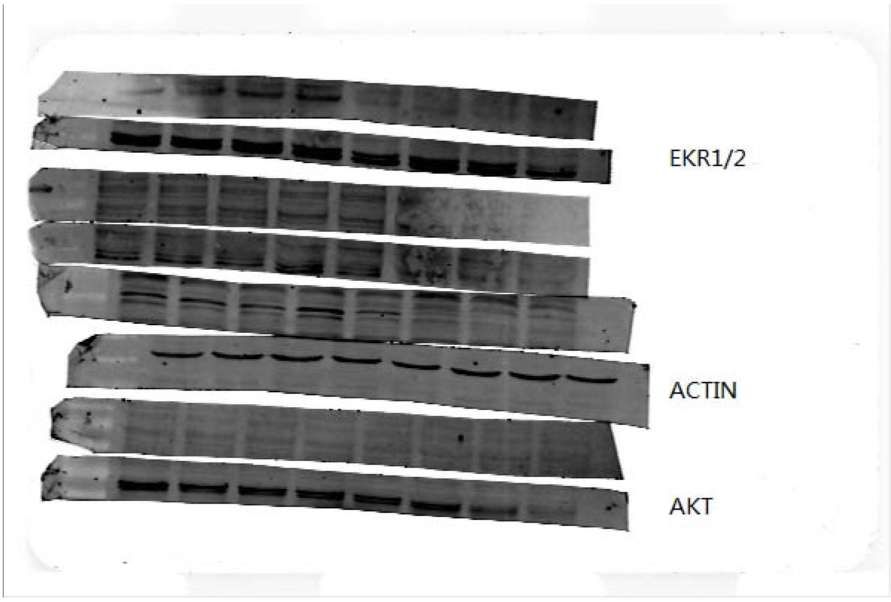

Supplement Supplement Figure S9: The blot is from Fig 3D in paper. MCF-7 cells were treated with **CPUY201112** at a concentration of 1 μM for different times. Then, the cells were collected and prepared for western blot analysis.

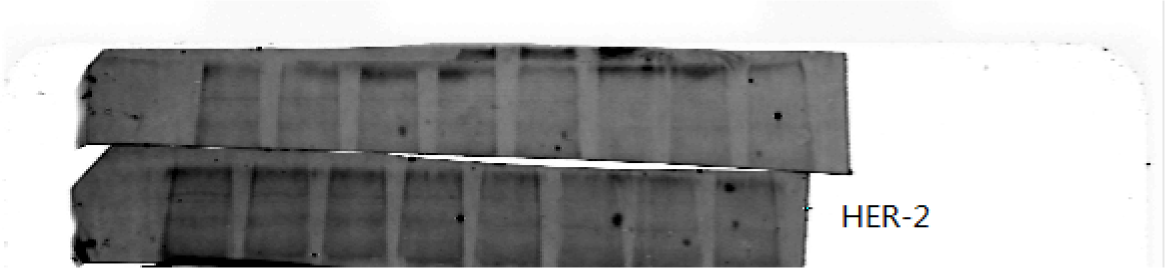

Supplement Figure S10: The blot is from Fig 3D in paper. MCF-7 cells were treated with **CPUY201112** at a concentration of 1 μM for different times. Then, the cells were collected and prepared for western blot analysis.

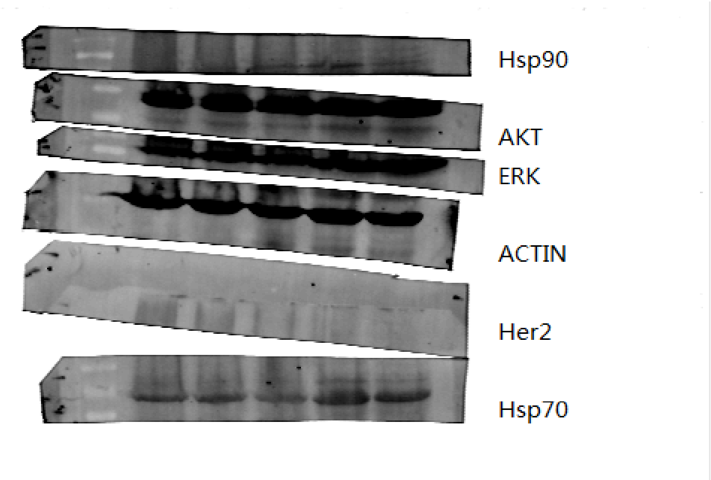

Supplement Figure S11: The blot is from Fig 6D in paper. Western blot analysis of Hsp70 and Akt in the tumor. *p<0.05; **p<0.01; Student *t*-test (n=3). The blots are cropped and the gel were run under the same condition.
